# Managing emotional and physical stress in sickle cell anemia: a review of effective strategies and approaches

**DOI:** 10.1097/MS9.0000000000002748

**Published:** 2025-03-05

**Authors:** Emmanuel Ifeanyi Obeagu, Getrude Uzoma Obeagu

**Affiliations:** a Department of Biomedical and Laboratory Science, Africa University, Zimbabwe; b School of Nursing Science, Kampala International University, Kampala, Uganda

**Keywords:** coping strategies, emotional stress, physical stress, sickle cell anemia, stress management

## Abstract

Sickle cell anemia (SCA) is a genetic blood disorder characterized by recurrent pain episodes, chronic complications, and significant emotional and physical stress. This review article explores effective strategies for managing both the emotional and physical aspects of stress in SCA patients. A comprehensive literature search was conducted across multiple databases, including PubMed, Scopus, and Google Scholar, using keywords such as “sickle cell anemia”, “stress management”, “psychological support”, and “pain management”. Emotional stress in SCA arises from chronic pain, frequent hospitalizations, and disease uncertainty, leading to conditions such as anxiety and depression. Effective management of emotional stress involves a combination of psychological counseling, cognitive-behavioral therapy (CBT), and support groups, which help patients develop coping strategies and address the mental health challenges of living with a chronic illness. This review evaluates various psychological interventions and their impact on patient outcomes, emphasizing the need for integrated mental health support in the management of SCA. Physical stress in SCA is primarily due to acute vaso-occlusive crises and chronic pain, which require effective pain management and preventive measures. The review explores pharmacological treatments, such as opioids and hydroxyurea, as well as nonpharmacological approaches, including physical therapy and lifestyle modifications. Additionally, the article discusses innovative therapies like gene therapy and stem cell transplantation, which hold promise for long-term disease management

## Introduction

Sickle cell anemia (SCA) is a chronic, genetic blood disorder characterized by the production of abnormal hemoglobin, which leads to the formation of sickle-shaped red blood cells. These deformed cells cause a range of complications, including vaso occlusive crises, chronic pain, anemia, and organ damage^[1,2]^. The physical manifestations of SCA, such as recurrent pain episodes and severe complications, are well-documented; however, the emotional and psychological stress experienced by patients is equally significant but less frequently addressed in clinical practice. SCA’s pathophysiology involves the substitution of valine for glutamic acid in the hemoglobin β-globin chain, resulting in hemoglobin S (HbS). Under low oxygen conditions, HbS aggregates into long, rigid polymers that distort red blood cells into a sickle shape. This distortion leads to obstruction of blood flow in small vessels, causing pain and acute complications such as stroke, acute chest syndrome, and organ damage. The chronic nature of these complications places a continuous emotional and physical burden on patients, necessitating effective stress management strategies to mitigate the impact of the disease^[3-5]^. The emotional burden of SCA includes psychological distress stemming from chronic pain, frequent hospitalizations, and the uncertainty of disease progression^[6]^. Many patients experience anxiety, depression, and a sense of helplessness due to the unpredictable nature of pain crises and the ongoing need for medical care. Studies have shown that this emotional distress can significantly affect patients’ adherence to treatment regimens, their relationships, and their overall quality of life. Addressing these emotional aspects through psychological support and counseling is essential for a holistic approach to managing SCA. Physical stress in SCA manifests primarily through acute vasoocclusive crises, which are episodes of severe pain resulting from the obstruction of blood flow in small vessels^[7]^. These crises are often managed with analgesics and supportive care, but their unpredictable nature poses a constant challenge for patients. Effective physical stress management requires a combination of pharmacological treatments, such as opioids and NSAIDs, and nonpharmacological approaches like physical therapy and lifestyle modifications. A thorough understanding of these methods is essential for developing effective treatment plans. Recent advancements in the management of SCA include novel therapeutic approaches such as hydroxyurea, which has been shown to reduce the frequency of pain crises and improve patient outcomes^[8]^. Hydroxyurea works by increasing fetal hemoglobin levels, which reduces the sickling of red blood cells and the resulting vaso-occlusive events. Additionally, emerging treatments like gene therapy and stem cell transplantation offer potential for long-term disease modification and stress reduction. These innovative therapies represent significant advancements in the care ofSCA patients, though their availability and application are still evolving. The integration of psychological support into SCA management is a relatively recent development, with increasing recognition of the need for mental healthcare in chronic illness management^[9]^. Psychological interventions such as cognitive-behavioral therapy (CBT) and support groups provide patients with tools to manage the emotional stress associated with SCA. CBT helps patients develop coping mechanisms for dealing with chronic pain and psychological distress, while support groups offer emotional support and a sense of community. These approaches are essential for addressing the emotional aspects of SCA management and improving overall patient care.

**Highlights**

- Emotional stress significantly impacts quality of life in sickle cell anemia patients.
- Integrated care approaches enhance both emotional wellbeing and physical health.
- Psychological support, including therapy and support groups, is essential for stress management.
- Effective pain management reduces physical stress and improves overall outcomes.
- Future research should focus on comprehensive strategies combining emotional and physical health interventions

Complementary therapies are another area of interest in managing stress in SCA patients^[10]^. Techniques such as acupuncture, massage therapy, and mindfulness-based stress reduction (MBSR) offer additional avenues for pain relief and stress management. Research indicates that these therapies can be effective in reducing pain, alleviating stress, and improving patients’ quality of life when used alongside conventional treatments. Exploring these complementary approaches helps to broaden the scope of stress management strategies available to SCA patients. Patient education and self-care strategies are fundamental components of SCA management^[11]^. Educating patients about the disease, treatment options, and self-care techniques empowers them to take an active role in their healthcare. Self-care practices include recognizing early signs of pain crises, maintaining hydration, and implementing stress-reduction techniques. Effective patient education fosters better disease management and encourages patients to adhere to treatment plans, ultimately improving their quality of life^[12-14]^. A multidisciplinary approach to SCA care involves collaboration among healthcare professionals, including hematologists, pain specialists, psychologists, and social workers. This approach ensures that all aspects of a patient’s well-being are addressed through coordinated care plans that incorporate medical treatments, psychological support, and patient education. Effective communication and teamwork among these professionals are crucial for providing comprehensive care and managing the emotional and physical stress of SCA^[15]^.

## Aim

The aim of this review article is to comprehensively explore and evaluate thevarious strategies and approachesfor managingboth the emotional and physical stress associated with sickle cell anemia (SCA).

## Rationale

Sickle cell anemia (SCA) is a complex, chronic genetic disorder characterized by a range of debilitating symptoms that impact both the emotional and physical aspects of patients’ lives. The disease, which results from a mutation in the hemoglobin gene, leads to the production of abnormally shaped red blood cells that cause recurrent pain, organ damage, and a reduced quality of life. Given the multifaceted nature of SCA, there is a pressing need to address the diverse challenges faced by patients through a comprehensive and multidisciplinary approach. This review article aims to fill a crucial gap in the current literature by exploring effective strategies for managing the emotional and physical stresses of SCA, which are often interconnected and require a holistic approach for optimal care. Emotional stress is a significant yet often under-recognized aspect of SCA management. Patients with SCA frequently experience psychological distress due to thechronic nature of the disease, frequent hospitalizations, and the impact of the disease on their daily lives. High levels of anxiety, depression, and stress can exacerbate physical symptoms, diminish treatment adherence, and impair overall wellbeing. Despite this, there is a relative scarcity of research focused on psychological support and emotional care for SCA patients. This review seeks to address this gap by evaluating existing therapeutic interventions and identifying best practices for psychological support, thus providing a foundation for improving emotional care in SCA.

In addition to emotional challenges, physical stress related to pain management represents another critical area of concern for SCA patients. The acute pain episodes and chronic pain associated with SCA significantly affect patients’ quality of life and complicate disease management. Traditional pain management approaches, such as opioid medications and pain relief techniques, have limitations and are often accompanied by side effects and issues of treatment adherence. There is a growing need for innovative pain management strategies that are effective, safe, and personalized to individual patient needs. The role of integrative medicine and complementary therapies in managing SCA is another underexplored area of research. While conventional treatments are crucial for managing SCA, there is increasing interest in how complementary therapies—such as acupuncture, meditation, and nutritional supplements—can support conventional treatments and offer additional benefits. These therapies have been shown to provide relief fromvarious health conditions, but their specific effectiveness for SCA patients remains inadequately studied. This review aims to assess the current evidence on these complementary therapies and their potential role in a comprehensive care strategy for SCA.

A multidisciplinary care approach is essential for addressing the complex needs of SCA patients. However, there is often a lack of integration among different healthcare disciplines in the management of SCA. This review emphasizes the importance of a collaborative care model that brings together hematologists, psychologists, nurses, and other specialists to provide holistic care. By exploring effective care models and highlighting successful examples of interdisciplinary collaboration, this review seeks to offer insights into how such models can be developed and implemented to improve patient outcomes. The review also addresses research gaps and future directions in SCA care. While advancements in treatment and management strategies are ongoing, there is a need for continued research to explore new therapies, refine existing treatment protocols, and investigate innovative approaches to patient care.

## Review methodology

The methodology employed in this review was designed to comprehensively examine and synthesize existing research on the management ofemotional andphysicalstress insickle cell anemia (SCA). This narrative review aimed to identify effective strategies and approaches for addressing these challenges and to provide a thorough evaluation of current practices and future directions for research. The review process followed a structured approach encompassing literature search, study selection, data extraction, quality assessment, and evidence synthesis.

## Literature search strategy

The literature search was conducted through a systematic and comprehensive search of multiple academic databases, including PubMed, Google Scholar, Scopus, and Web of Science. The search strategy employed a combination of relevant keywords and phrases related to the review’s focus. Terms such as “Sickle Cell Anemia”, “emotional stress”, “physical stress”, “pain management”, “psychological support”, “integrative medicine”, and “multidisciplinary care” were used to identify studies and reviews relevant to the management of SCA. The search was limited to peer-reviewed articles published in English between January 2000 and December 2023 to ensure the inclusion of the most recent and relevant research.

## Study selection process

Study selection involved a multistep process to ensure that only the most relevant and high-quality studies were included in the review. Initially, titles and abstracts of articles retrieved from the databases were screened based on predefined inclusion criteria, such as relevance to emotional and physical stress in SCA, focus on management strategies, and evidence of effectiveness. Studies that met these criteria were then subjected to a full-text review. Exclusion criteria included studies that were not directly related to SCA, focused on unrelated health conditions, or lacked a rigorous methodological approach. This process ensured that the selected studies provided valuable insights into the management of SCA-related stress.

## Data extraction and analysis

Data extraction was carried out to systematically collect relevant information from the selected studies. A standardized data extraction form was used to capture key details from each study, including studyobjectives, methods, keyfindings,and evidence of effectiveness for various management strategies. The data was then analyzed to identify common themes, patterns, and gaps in the current knowledge base. This involved summarizing the effectiveness of psychological interventions, pain management techniques, integrative therapies, and multidisciplinary care models. The analysis also included a review of the methods used in the studies to assess the strength and reliability of the evidence.

## Quality assessment of included studies

Each study was evaluated using established criteria for assessing study quality, such as the Critical Appraisal Skills Program (CASP) checklist for qualitative studies and the Jadad Scale for quantitative studies. These tools helped to evaluate the studies’ validity, reliability, and applicability to the review’s objectives. Studies that met high-quality standards were given more weight in the review’s conclusions, ensuring that the recommendations were based on robust and credible evidence. A total number of 68 papers were consulted.

## Emotional stress in sickle cell anemia

Sickle cell anemia (SCA) imposes significant emotional stress on patients due to the chronic and unpredictable nature of the disease^[16]^. This genetic blood disorder leads to recurrent pain episodes, frequent hospital visits, and ongoing health complications, which collectively contribute to a substantial emotional burden. Patients with SCA often experience anxiety related to the unpredictability of pain crises, a constant worry about future health complications, and the potential for disease progression. Additionally, the visibility of SCA symptoms, such as pain and fatigue, can lead to feelings of stigma and isolation, further exacerbating emotional distress. This section explores these emotional stressors in detail and their profound impact on patients’ mental health. Psychological distress in SCA manifests primarily through anxiety and depression, which are prevalent among patients due to the chronic and severe nature of the disease^[17]^. Anxiety often arises from concerns about the future, fear of pain crises, and the management of ongoing medical treatments. Depression, characterized by persistent feelings of sadness, hopelessness, and loss of interest, is also common and can be triggered by the cumulative effects of living with a chronic illness. Research indicates that patients with SCA are at a higher risk of experiencing these mental health conditions compared to the general population. Effective management of these emotional challenges is crucial for improving overall patient well-being and adherence to treatment regimens.

Emotional stress significantly affects treatment adherence and quality of life for individuals with SCA^[18]^. High levels of psychological distress can lead to nonadherence to treatment plans, missed medical appointments, and a decreased willingness to engage inself-care activities. Thisnonadherencecan result in more frequent pain crises, hospitalizations, and complications. Additionally, emotional stress negatively impacts patients’ quality of life by reducing their ability to participate in daily activities, affecting their relationships, and diminishing their overall sense of well-being. Addressing these emotional stressors through targeted interventions is essential for improving treatment outcomes and enhancing the quality of life for SCA patients. To manage emotional stress in SCA, several psychological interventions have been shown to be effective^[19]^. Cognitive-behavioral therapy (CBT) is one such intervention that helps patients develop coping strategies for managing chronic pain, reducing anxiety, and addressing depressive symptoms. CBT focuses on identifying and altering negative thought patterns and behaviors, helping patients develop healthier coping mechanisms and improving their emotional resilience. Support groups offer another valuable resource, providing patientswitha sense ofcommunity and sharedexperiences, which can reduce feelings of isolation and offer practical advice from peers who understand the challenges of living with SCA.

Family and social support play a critical role in managing emotional stress in SCA patients^[20]^. Support from family members can provide emotional comfort, practical assistance with daily tasks, and encouragement to adhere to treatment regimens. Social support, including relationships with friends, community resources, and involvement in SCA support networks, also contributes to emotional well-being. Studies have demonstrated that strong family support and positive social relationships are associated with better mental health outcomes for SCA patients^[21,22]^. Encouraging patients to build and maintain supportive relationships is a key component of effective emotional stress management. The stigma associated with SCA can exacerbate emotional stress by causing patients to feel marginalized or misunderstood^[23]^. Addressing stigma involves increasing awareness about SCA and promoting education to reduce misconceptions. Building resilience is also an important strategy for managing emotional stress. Resilience training helps patients develop the psychological strength to cope with the challenges of living with a chronic illness. Programs designed to foster resilience often include techniques for stress management, problemsolving, and goal-setting, which can empower patients to face the difficulties of SCA with greater confidence and optimism. Integrated care models that incorporate psychological support into routine medical care are essential for managing emotional stress in SCA patients^[24,25]^. These models involve collaboration among hematologists, psychologists, social workers, and other healthcare professionals to provide a holistic approach to patient care. By integrating mental health services with medical treatments, these models address both the physical and emotional aspectsofSCA,leadingtomorecomprehensiveandeffectivecare. This collaborative approach ensures that patients receive the support they need to manage their disease and its emotional impact. Patient education is a fundamental component of emotional stress management for SCA patients. Educating patients about their disease, treatment options, and self-care techniques empowers them to manage their health more effectively and reduces anxiety relatedto disease management. Education should cover topics such as understanding disease progression, recognizing signs of complications, and utilizing stress-reduction techniques. Providing patients with accurate information and practical skills helps them navigate the challenges of SCA and fosters a sense of control over their condition.

## Physical stress and pain management

Physical stress in sickle cell anemia (SCA) predominantly arises from the frequent and severe vaso-occlusive crises that patients experience^[26]^. These crises occur when sickle-shaped red blood cells obstruct blood flow in small vessels, causing acute pain, tissue damage, and various complications such as stroke, acute chest syndrome, and organ damage. This chronic cycle of pain and crisis management leads to significant physical stress for patients, affecting their daily functioning, quality of life, and overall health. Understanding the nature of this physical stress is essential for developing effective pain management strategies and improving patient outcomes. Pharmacological treatments are the cornerstone of pain management for SCA patients experiencing vaso-occlusive crises^[27]^. Opioids, such as morphine and hydrocodone, are commonly used for managing moderate to severe pain. NSAIDs like ibuprofen and naproxen are also employed to alleviate mild to moderate pain and reduce inflammation.

Hydroxyurea, a disease-modifying therapy, can reduce the frequency of pain crises by increasing fetal hemoglobin levels, which decreases red blood cell sickling. Each of these treatments has its benefits and potential side effects, making it crucial to tailor pain management strategies to individual patient needs and regularly adjust treatment plans based on efficacy and tolerability. In addition to pharmacological treatments, nonpharmacological approaches play a significant role in managing physical stress and pain in SCA^[27]^. Techniques such as heat therapy, hydration, and physical therapy can complement medical treatments and offer additional relief.Heat therapy,includingwarm bathsandheating pads, helps relax muscles and improve blood flow. Adequate hydration is essential for preventing sickle cell crises and managing pain. Physical therapy, including gentle exercises and stretching, can improve mobility and reduce pain. Integrating these nonpharmacological approaches into treatment plans can provide patients with a more comprehensive pain management strategy.

Patient education is a crucial component of effective pain management for SCA patients. Educating patients about recognizing early symptoms of a vaso-occlusive crisis, adhering to prescribed treatments, and implementing self-care practices is essential for managing physical stress. Patients should be informed about the importance of hydration, the benefits of regular exercise, and strategies for managing pain at home. Educationalprogramsthatprovideclearinstructionsandsupport can empower patients to take an active role in their care and reduce the frequency and severity of pain crises^[24]^. Recent advancements in pain management for SCA patients have introduced innovative therapies that offer new options for pain relief and stress management. New pharmacological agents, such as novel opioids and nonopioid analgesics, are being explored for their efficacy in managing chronic pain. Additionally, research into gene therapy and stem cell transplantation offers potential long-term solutions for reducing the incidence of vaso-occlusive crises and improving patient outcomes. These advancements represent promising future directions for managing physical stress in SCA and enhancing the effectiveness of treatment regimens^[25]^. Psychological approaches, such as cognitive-behavioral therapy (CBT), can be integrated into pain management strategies to help patients cope with chronic pain and reduce physical stress. CBT techniques focus on altering negative thought patterns related to pain and teaching patients effective coping strategies. Mindfulness and relaxation techniques, including meditation and deep breathing exercises, are also used to help patients manage pain and reduce stress. These psychological interventions can complement medical treatments and provide patients with tools to manage pain more effectively. Preventing vaso-occlusive crises is a key goal in managing physical stress for SCA patients^[28]^. Prevention strategies include regular use of hydroxyurea, vaccination against infections, and avoidance of known triggers such as dehydration, extreme temperatures, and high altitudes. By implementing these strategies, patients can reduce the frequency of crises and the associated physical stress. Adherence to preventive measures and regular follow-up care are essential for maintaining effective disease management and minimizing the impact of SCA on patients’ physical health.

Multidisciplinary care teams, which include hematologists, pain specialists, physiotherapists, and psychologists, are essential for providing comprehensive pain management for SCA patients^[24]^. These teams work collaboratively to address all aspects of pain management, from pharmacological treatments to psychological support and physical therapy. Effective communication and coordination among team members ensure that patients receive a well-rounded approach to managing their pain and physical stress. This collaborative model facilitates the development of individualized treatment plans and improves overall patient care. Complementary therapies offer additional options for managing pain and physical stress in SCA patients. Techniques such as acupuncture, massage therapy, and aromatherapy can provide symptomatic relief and enhance the effectiveness of conventional treatments. These therapies work by targeting pain pathways, reducing muscle tension, and promoting relaxation. While more research is needed to fully understand their benefits, complementary therapies are a valuable addition to traditional pain management strategies and can offer patients more options for managing their condition.

## Psychological support and counseling

Sickle cell anemia (SCA) is a chronic and debilitating condition that not only affects patients physically but also takes a significant emotional and psychological toll. The nature of SCA, with its recurrent pain crises, chronic complications, and uncertainty about disease progression, can lead to severe psychological distress^[29]^. Patients frequently experience anxiety, depression, and a sense of helplessness, which can negatively impact their adherence to treatment and overall quality of life. Psychological support and counseling are essential components of comprehensive SCA care, aimed at addressing these emotional challenges and helping patients develop effective coping mechanisms. Cognitive-behavioral therapy (CBT) is a well-established psychological intervention used to address emotional distress in SCA patients. CBT focuses on identifying and modifying negative thought patterns and behaviors related to chronic pain and illness. Through structured sessions, patients learn to recognize unhelpful thoughts, develop healthier cognitive patterns, and implement effective coping strategies. CBT can help patients manage anxiety and depression, improve their emotional resilience, and enhance their ability to handle the daily challenges of living with SCA. Research has demonstrated that CBT is effective in reducing pain perception, improving mood, and increasing patients’ engagement in self-care practices^[30]^. Support groups offer a vital resource for individuals with SCA, providing emotional support, shared experiences, and practical advice. These groups facilitate peer interactions, where patients can share their experiences, offer and receive emotional support, and learn from others who understand the unique challenges of living with SCA. Support groups can also serve as a platform for education on disease management and coping strategies. Studies have shown that participation in support groups is associated with improved psychological well-being, increased treatment adherence, and a stronger sense of community among patients.

Integrating psychological services into routine SCA care is a strategy for addressing the emotional needs of patients in a structured and consistent manner^[31]^. This approach involves incorporating mental health professionals, such as psychologists or clinical social workers, into the care team alongside hematologists and other specialists. By providing regular access to psychological support, this model ensures that patients receive comprehensive care that addresses both their physical and emotional health. Integrated care models have been shown to improve patient outcomes by fostering a collaborative environment where mental health issues are addressed proactively. Effective counseling services for SCA patients involve a range of strategies tailored to individual needs^[32]^. These strategies include establishing a therapeutic alliance, setting achievable goals, and using evidence-based techniques to address specific psychological issues. Counselors should be trained in managing chronic illness and skilled in techniques such as motivational interviewing, which helps patients explore and resolve ambivalence about behavior change. Personalizing counseling approaches to fit each patient’s unique situation and preferences enhances the effectiveness of the support provided.

Anxiety and depression are common among SCA patients and require targeted psychological interventions for effective management^[33]^. Interventions such as relaxation training, mindfulness-based stress reduction (MBSR), and psychoeducation can be used to address symptoms of anxiety and depression. Relaxation training techniques, including progressive muscle relaxation and deep breathing exercises, help patients manage stress and reduce anxiety. MBSR programs teach patients mindfulness techniques to stay present and reduce the impact of negative thoughts and feelings. Psychoeducation provides patients with information about the relationship between their mental health and chronic illness, helping them understand and manage their symptoms. Psychoeducation plays a crucial role in empowering SCA patients to manage their emotional well-being and adhere to treatment plans^[34]^. By providing patients with knowledge about their condition, treatment options, and coping strategies, psychoeducation fosters a sense of control and selfefficacy. Educated patients are better equipped to understand their symptoms, engage in effective self-care, and make informed decisions about their health. Psychoeducation also helps patients recognize the importance of seeking psychological support and utilizing available resources to manage their emotional and physical stress. Building resilience is a key goal of psychological support programs for SCA patients^[35]^. Resilience training programs focus on helping patients develop the skills to adapt to the challenges of living with a chronic illness. These programs often include components such as stress management techniques, problem-solving skills, and strategies for maintaining a positive outlook. By enhancing patients’ ability to cope with adversity, resilience training supports their emotional well-being and helps them navigate the difficulties associated with SCA. Evidence suggests that resilience training can improve patients’ psychological health and their ability to manage their disease. Evaluating the effectiveness of psychological interventions is essential for ensuring that support services meet the needs of SCA patients^[36]^. This evaluation involves assessing the impact of various interventions on patients’ emotional health, treatment adherence, and overall quality of life. Methods for evaluating effectiveness include quantitative measures, such as standardized psychological assessments and patient-reported outcomes, as well as qualitative feedback from patients about their experiences with psychological support services. Regular evaluation helps identify successful approaches and areas for improvement in psychological care for SCA patients.

## Innovative therapeutic approaches

Gene therapy represents a groundbreaking approach to treating sickle cell anemia (SCA) by targeting the root cause of the disease at the genetic level^[37]^. Recent advancements have focused on techniques such as CRISPR-Cas9 gene editing to correct the mutation in the hemoglobin gene responsible for sickling. In clinical trials, CRISPR-Cas9 has been used to edit the β-globin gene to produce normal hemoglobin, aiming to cure SCA or significantly reduce symptoms. Early results from these trials are promising, with some patients showing sustained reductions in disease symptoms and improved quality of life. Gene therapy offers the potential for long-term remission or even a functional cure for SCA, representing a major shift in the management of this chronic condition. Stem cell transplantation is a well-established but continually evolving therapeutic approach for SCA, offering the potential for a cure through hematopoietic stem cell replacement^[38]^. This treatment involves replacing the patient’s defective hematopoietic cells with healthy stem cells from a matched donor, typically a sibling or an unrelated donor. Recent advances in stem cell transplantation techniques, such as improved conditioning regimens and the development of less toxic preparatory treatments, have increased the feasibility and success rates of this approach. New strategies are also being explored, including the use of gene-edited stem cells and alternative sources of stem cells, such as umbilical cord blood. These innovations aim to make stem cell transplantation a viable option for a broader range of SCA patients.

While hydroxyurea remains a cornerstone of SCA treatment for its ability to increase fetal hemoglobin levels and reduce sickling, there is a strong push for developing new drugs to offer additional treatment options^[39]^. Recent advancements have introduced several novel agents, such as crizanlizumab and voxelotor, which target different aspects of SCA pathology. Crizanlizumab is a monoclonal antibody that inhibits the interaction between sickle cells and blood vessel walls, reducing the frequency of pain crises. Voxelotor increases hemoglobin’s affinity for oxygen, preventing sickling, and improving red blood cell health. These new drugs represent significant progress in providing more targeted and effective treatments for managing SCA. Hemoglobin F (HbF) induction therapies are a promising avenue for the management of SCA^[40]^. Hemoglobin F, the fetal form of hemoglobin, inhibits the sickling process and is associated with fewer disease symptoms in SCA patients. Research intodrugsthat can induce HbF production, such as decitabine and butyrate derivatives, has shown potential for reducing disease severity. These therapies work by reactivating the fetal hemoglobin gene, offering a less invasive alternative to more aggressive treatments. Ongoing clinical trials are investigating the long-term efficacy of these therapies and their potential to become a standard treatment option for SCA.

Innovative approaches in pain management are continually being explored to address the complex pain experiences of SCA patients^[41]^. Techniques such as virtual reality (VR) for pain distraction, biofeedback, and transcranial magnetic stimulation (TMS) are being investigated for their efficacy in managing chronic pain. VR offers immersive experiences that can distract patients from pain, while biofeedback trains patients to control physiological responses to pain. TMS, a noninvasive neuromodulation technique, is being studied for its potential to alter pain perception pathways. These emerging technologies represent a new frontier in managing the physical stress and pain associated with SCA. Personalized medicine approaches are transforming the treatment landscape for SCA by tailoring therapies to individual patients based on their genetic profiles, disease characteristics, and treatment responses^[42]^. Advances in genomic sequencing and bioinformatics allow for the identification of patient-specific mutations and the development of targeted therapies. Personalized medicine strategies include customizing drug regimens, optimizing dosing protocols, and identifying suitable candidates for advanced therapies such as gene editing or stem cell transplantation. This approach aims to improve treatment efficacy and reduce adverse effects by aligning interventions with each patient’s unique needs.

Vaccination strategies have gained attention for their role in preventing infections and managing complications in SCA patients^[43]^. New vaccines and booster regimens targeting pathogens that commonly affect SCA patients, such as pneumococcus, Haemophilus influenzae, and meningococcus, are being developed. These vaccines aim to reduce the incidence of infections that can trigger pain crises or lead to severe complications. Additionally, research is exploring the effectiveness of conjugate vaccines and new adjuvants to enhance immune responses in individuals with SCA. These advancements are expected to improve disease management and prevent infection-related complications. Combination therapies are a burgeoning area of research in SCA treatment, involving the use of multiple therapeutic agents to address different aspects of the disease simultaneously^[44]^. Research is exploring combinations of existing drugs, such as hydroxyurea with crizanlizumab or voxelotor, as well as novel combinations of newer agents and complementary therapies. The goal of combination therapies is to enhance overall treatment efficacy by targeting various mechanisms of the disease and addressing both acute and chronic aspects of SCA. Early studies suggest that combination approaches could offer synergistic effects that improve patient outcomes more effectively than single-agent therapies.

## Integrative medicine and complementary therapies

Integrative medicine combines conventional medical treatments with complementary therapies to provide a holistic approach to managing chronic conditions like sickle cell anemia (SCA)^[45]^. This approach recognizes that managing SCA requires addressing not only the physical symptoms but also the emotional, psychological, and lifestyle factors affecting patient well-being. By integrating complementary therapies with standard medical care, patients can benefit from a more comprehensive treatment plan that aims to improve overall health, enhance quality of life, and support effective disease management. Integrative medicine emphasizes the collaboration between healthcare providers and patients to develop personalized care strategies that align with the patient’s values and preferences. Nutritional therapy is a crucial component of integrative medicine for SCA, aiming to optimize patients’ health through diet and nutritional supplements^[46]^. A well-balanced diet rich in essential nutrients can help manage symptoms and prevent complications. For example, folic acid is essential for red blood cell production, while iron is important for hemoglobin synthesis. Recent studies have also explored the benefits of omega-3 fatty acids and antioxidant-rich foods in reducing inflammation and improving overall health in SCA patients. Nutritional counseling helps patients make informed dietary choices and incorporate supplements that support their specific health needs.

Acupuncture and acupressure are traditional Chinese medicine practices that offer potential benefits for managing pain and other symptoms associated with SCA^[47]^. Acupuncture involves inserting fine needles into specific points on the body to stimulate energy flow and promote healing. Acupressure, a similar technique, uses finger pressure on specific points to achieve therapeutic effects. Both methods have been studied for their ability to reduce pain, alleviate stress, and improve quality of life in chronic illness. While more research is needed to fully establish their efficacy, these therapies provide additional options for pain management and symptom relief. Mindfulness-Based Stress Reduction (MBSR) and meditation techniques are effective complementary therapies for managing emotional and physical stress in SCA^[48]^. MBSR programs teach mindfulness meditation practices that help patients become more aware of their thoughts, emotions, and bodily sensations. Techniques such as deep breathing exercises, body scans, and guided imagery are used to reduce stress, manage pain, and improve emotional well-being. Research has demonstrated that MBSR can help patients with chronic illnesses cope with pain and stress, making it a valuable addition to standard SCA treatments. Yoga and Tai Chi are mind-body practices that offer multiple benefits for SCA patients, including physical exercise, stress reduction, and pain management^[49]^. Yoga involves a series of postures and breathing techniques designed to improve flexibility, strength, and relaxation. Tai Chi is a form of gentle martial arts that emphasizes slow, deliberate movements and deep breathing. Both practices have been shown to reduce pain, improve physical function, and enhance emotional well-being in chronic illness. Regular participation in yoga and Tai Chi can help SCA patients manage symptoms and improve their overall quality of life.

Herbal medicine and phytotherapy involve using plant-based substances for therapeutic purposes. In SCA, certain herbs and plant extracts are explored for their potential to alleviate symptoms and support treatment^[50]^. Ginger and turmeric, known for their anti-inflammatory properties, are commonly used to reduce pain and inflammation. Garlic and ginseng are also studied for their potential to enhance immune function and overall health. While these therapies are popular, it is important for patients to consult healthcare providers to ensure the safe and effective use of herbal remedies alongside conventional treatments. Biofeedback is a technique that teaches patients to control physiological processes such as muscle tension, heart rate, and blood pressure. By using real-time feedback from sensors, patients can learn to manage their body’s responses to stress and pain. In SCA, biofeedback can be used to reduce pain, manage stress, and improve relaxation. Studies have shown that biofeedback can be effective in helping patients with chronic pain conditions by increasing their awareness of physiological processes and teaching self-regulation techniques. Integrating complementary therapies into conventional SCA treatment plans requires careful consideration of the benefits and risks of each therapy^[51]^. A collaborative approach involving healthcare providers, patients, and complementary therapy practitioners ensures that all aspects of care are addressed. Integrative care plans might include combining pharmacological treatments with acupuncture, nutritional counseling, or mindfulness techniques. Effective integration involvesevaluatingevidenceforeachtherapy,consideringpatient preferences, and monitoring outcomes to adjust treatment plans as needed. Evaluating the effectiveness of complementary therapies for SCA involves assessing their impact on symptoms, quality of life, and overall patient well-being. Methods for evaluation include randomized controlled trials, systematic reviews, and patient-reported outcome measures. Research should focus on establishing the clinical efficacy of therapies like acupuncture, yoga, and herbal remedies through rigorous scientific methods. Effective evaluation helps determine which complementary therapies provide the most benefit for SCA patients and guides future research and clinical practice.

## Patient education and self-care strategies

Patient education is a fundamental aspect of managing sickle cell anemia (SCA), aiming to empower individuals with the knowledge and skills needed to take control of their health^[52]^. Effective patient education helps patients understand the nature of their disease, recognize symptoms, and adhere to treatment regimens. By providing clear, accessible information about SCA, healthcare professionals enable patients to make informed decisions about their care, manage their symptoms, and prevent complications. Education often covers topics such as disease pathophysiology, the role of medications, and strategies for managing pain and preventing crises. By equipping patients with this knowledge, educational programs aim to foster greater engagement in selfcare and improve overall health outcomes. Developing self-care skills is essential for individuals with SCA to effectively manage their condition on a daily basis^[53]^. Self-care encompasses a range of activities designed to maintain health, manage symptoms, and prevent complications. These skills include understanding medication regimens, recognizing early signs of complications, and implementing lifestyle changes that support well-being. For instance, patients are educated on the importance of staying hydrated, maintaining a balanced diet, and engaging in regular physical activity. Additionally, patients learn how to monitor their symptoms and when to seek medical help. These self-care strategies are vital for reducing the frequency and severity of pain crises, managing chronic symptoms, and maintaining a good quality of life. Health literacy is a key component of effective patient education programs. It refers to the ability of individuals to access, understand, and use health information to make informed decisions. Programs designed to enhance health literacy often include educational workshops, informational brochures, and interactive tools that simplify complex medical information. These resources help patients and their families understand SCA, including its causes, symptoms, and treatment options. By improving health literacy, patients become more capable of managing their disease, navigating the healthcare system, and engaging in preventive care measures. This empowerment is crucial for achieving better health outcomes and fostering patient autonomy in managing their condition.

Pain management is a critical aspect of SCA care, and educating patients on effective techniques is essential for alleviating suffering and improving quality of life^[54]^. Education often includes teaching patients about various methods for managing pain, such as medication use, heat application, and distraction techniques. Patients learn about the different types of pain associated with SCA and appropriate ways to manage each type. Techniques like deep breathing exercises, progressive muscle relaxation, and mindfulness meditation are also taught to help patients cope with pain. Additionally, patients are encouraged to develop a pain management plan that includes recognizing triggers, using medications as prescribed, and seeking medical assistance when necessary. Preventing sickle cell crises involves a combination of lifestyle modifications and proactive health measures^[4,25]^. Patients are educated about the importance of hydration, temperature regulation, and avoiding known triggers for pain crises. For example, staying hydrated helps maintain blood flow and reduce sickling, while avoiding extreme temperatures and strenuous activities can prevent crisis episodes. Education also includes information on vaccinations and regular medical check-ups to prevent infections and manage complications. By following these preventive strategies, patients can reduce the frequency of crises and improve their overall health. Adherence to treatment regimens is crucial for effective SCA management. Education focuses on helping patients understand the importance of taking medications as prescribed, attending medical appointments, and following up on recommended treatments. Techniques to support adherence include setting up medication reminders, using pill organizers, and creating a structured schedule for taking medications. Healthcare professionals also work with patients to address any barriers to adherence, such as side effects or difficulties with the medication regimen. Support systems, such as patient support groups and online resources, are also recommended to help patients stay motivated and informed about their treatment.

Support systems and resources are invaluable for SCA patients in managing their condition effectively^[55]^. These resources include support groups, online forums, and community organizations that provide information, emotional support, and practical advice. Support groups offer a platform for patients to share experiences, seek advice, and find encouragement from others facing similar challenges. Online forums and community organizations offer additional resources for educational materials, access to healthcare professionals, and opportunities for patient advocacy. Engagingwith thesesupport systems helps patientsfeel less isolated and more connected to a community of individuals who understand their experiences. Behavioral health approaches are effective for addressing the emotional challenges of living with SCA^[56]^. These approaches include cognitive-behavioral therapy (CBT), stress management techniques, and behavioral interventions designed to improve emotional well-being. Patients learn to recognize and change negative thought patterns, develop coping strategies for managing stress, and implement practical steps for improving mental health. Programs that focus on behavioral health help patients build resilience, manage anxiety and depression, and improve their overall emotional state. By addressing these emotional aspects of SCA, patients can enhance their ability to cope with the disease and improve their quality of life. Family and caregivers play a significant role in supporting SCA patients through education and care^[57]^. Training for family members and caregivers focuses on understanding the disease, supporting patient self-care, and recognizing signs of complications. Effective caregiver education programs include information on medication management, symptom monitoring, and emotional support techniques. By involving family members in the education process, patients receive additional support and encouragement, which is essential for maintaining adherence to treatment plans and managing the emotional challenges of SCA. Assessing and adjusting patient education programs ensures that educational initiatives meet the evolving needs of SCA patients. Regular evaluation of educational materials, feedback from patients, and outcomes of educational interventions help identify areas for improvement. Methods for assessment include surveys, focus groups, and interviews with patients and healthcare providers. This ongoing evaluation process helps refine educational strategies, develop newresources, and ensure that patients receive up-to-date and effective information for managing their condition.

## Multidisciplinary care teams

Multidisciplinary care teams represent a collaborative approach to managing sickle cell anemia (SCA), integrating the expertise of various healthcare professionals to address the complex needs of patients^[58]^. These teams consist of specialists from diverse fields, including hematology, nursing, psychology, social work, and nutrition, among others. Each member of the team brings a unique set of skills and knowledge, working together to provide comprehensive, patient-centered care. This team-based approach ensures that all aspects of SCA management—ranging from medical treatments to emotional support—are addressed in a coordinated manner. By combining expertise from different disciplines, these teams aim to improve patient outcomes and enhance the overall quality of care. In a multidisciplinary care team, each healthcare professional has a specific role that contributes to the overall management of SCA^[59]^. Hematologists lead the clinical management of SCA, overseeing treatment plans, managing complications, and prescribing medications. Nurses provide patient education, administer treatments, and offer ongoing support for managing symptoms and side effects. Psychologists and counselors address the emotional and psychological challenges of living with SCA, offering therapy for anxiety, depression, and coping strategies. Social workers assist with navigating healthcare resources, advocating for patient needs, and providing support for practical issues such as insurance and financial concerns. Dietitians offer nutritional counseling to help patients maintain a balanced diet and manage health conditions related to SCA. The multidisciplinary approach offers numerous benefits for managing SCA. By bringing together experts from various fields, this approach ensures that patients receive comprehensive care that addresses both their medical and non-medical needs. One significant benefit is the holistic management of SCA, where the physical, emotional, and social aspects of the disease are all considered. This approach leads to better disease management, as team members collaborate to develop and implement treatment plans that are more effective than what any single provider could achieve alone. Additionally, the collaborative nature of the team fosters communication between different specialists, which help stop revent errors, streamline care processes, and ensure that patients receive consistent and cohesive treatment^[58]^.

Integrated care models are designed to improve patient outcomes by coordinating care across multiple disciplines. These models focus on creating a seamless care experience for patients, where different aspects of their care are interconnected and managed as a whole. For SCA patients, integrated care models involve regular team meetings, shared medical records, and joint care planning sessions. These practices ensure that all team membersare awareofthepatient’scondition,treatment progress, and any changes in care plans. By integrating care, these models help to address complex medical needs, enhance patient satisfaction, and improve health outcomes. Research has shown that integrated care models can lead to more effective management of chronic conditions and better patient experiences. Patient-centered care is a core principle of multidisciplinary teams, focusing on the needs, preferences, and values of patients. This approach ensures that patients are active participants in their own care, with team members working to address their specific concerns andgoals. In SCA management, patient-centered care involves involving patients in decision-making processes, listening to their experiences, and adapting care plans based on their feedback. The multidisciplinary team supports patient-centered care by offering a range of services tailored to individual needs, including emotional support, educational resources, and practical assistance. This approach helps to build trust between patients and healthcare providers and ensures that care is both effective and empathetic^[59]^.

Managing chronic diseases like SCA requires ongoing, comprehensive care, which is well-suited to a multidisciplinary team approach. These teams are equipped to handle the long-term aspects of SCA management, including routine check-ups, crisis intervention, and preventive care. Regular team meetings and coordinated care plans ensure that all aspects of the patient’s health are monitored and managed effectively. By maintaining a focus on both immediate needs and long-term health, multidisciplinary teams help patients manage their condition over time, reduce the frequency of crises, and improve overall health outcomes. This approach also supports proactive care, where potential issues are addressed before they become serious problems. Effective communication is essential for the success of multidisciplinary care teams. Strategies for improving communication include establishing regular team meetings, using shared electronic health records, and creating clear protocols for information sharing. Team members must communicate openly about patient needs, treatment plans, and any concerns that arise. Interdisciplinary collaboration also involves setting common goals for patient care and working together to achieve these objectives. By fostering open communication and collaboration, teams can ensure that all members are aligned in their approach to patient care, leading to more effective management of SCA^[58]^. Despite the benefits, multidisciplinary care teams face several challenges in managing SCA. These challenges include coordination issues, where communication breakdowns between team members can lead to inconsistencies in patient care. There may also be resource limitations, where access to certain specialists or services is restricted due to cost or availability. Additionally, conflicts between team members can arise from differing opinions on treatment approaches or priorities. Addressing these challenges requires proactive measures, such as implementing structured communication systems, seeking additional resources, and fostering a culture of mutual respect and collaboration among team members. Evaluating the effectiveness of multidisciplinary care teams is crucial for ensuring that these teams provide high-quality care for SCA patients. Evaluation methods include patient satisfaction surveys, outcome assessments, and team performance reviews. These evaluations help to identify areas for improvement, measure the impact of care interventions, and ensure that patient needs are being met. Effective evaluation involves collecting and analyzing data on various aspects of care, including patient outcomes, team dynamics, and the efficiency of care processes. By regularly assessing team performance, healthcare organizations can makenecessary adjustments to improve care delivery and patient experiences.

## Future directions and research opportunities

### Advancing novel therapies for sickle cell anemia

One of the most promising future directions in sickle cell anemia (SCA) management is the development and implementation of novel therapies^[60]^. Recent advancements in gene therapy and gene editing technologies offer new hope for curing SCA at a fundamental level. Techniques such as CRISPR-Cas9 gene editing have shown potential in correcting the genetic mutations responsible for SCA. Clinical trials are exploring these innovative approaches, with some showing promising results in preclinical and early-phase studies. Additionally, research into stem cell therapies aims to develop effective methods for replacing defective blood cells with healthy ones. These emerging therapies represent a significant shift from traditional management strategies and could potentially provide long-term solutions for SCA patients.

### Exploring integrative approaches for comprehensive care

Integrative approaches are becoming increasingly relevant in SCA management, combining conventional treatments with complementary therapies to provide holistic care^[61]^. Research is needed to evaluate the effectiveness of integrative medicine practices, such as acupuncture, meditation, and nutritional supplements, in managing SCA symptoms and improving quality of life. Integrative care models aim to address both physical and emotional aspects of the disease, providing patients with a comprehensive care experience. Future research opportunities include conducting large-scale studies to assess the benefits and safety of these complementary therapies, as well as exploring how these practices can be effectively incorporated into standard care regimens.

### Innovative therapies: gene therapy and stem cell transplantation

In addition to traditional and complementary therapies, innovative treatments such as gene therapy and stem cell transplantation are becoming essential components of integrative care. These cutting-edge approaches aim to address the root causes of diseases and improve patient outcomes significantly.

### Gene therapy

Gene therapy involves modifying or manipulating genes to treat or prevent diseases, particularly genetic disorders. This innovative approach holds the potential to correct underlying genetic defects, offering hope for conditions previously deemed incurable. Gene therapy can be administered through various techniques, including delivering therapeutic genes via viral vectors, editing genes using CRISPR-Cas9 technology, or replacing defective genes with functional copies. These methods can restore normal function in cells, potentially halting or reversing disease progression. Gene therapy has shown promise in treating inherited disorders such as cystic fibrosis, hemophilia, and certain types of muscular dystrophy. Clinical trials have demonstrated encouraging results, with patients experiencing significant improvements in their health and quality of life. For example, the recent approval of gene therapy for spinal muscular atrophy has revolutionized treatment options for affected individuals. Despite its potential, gene therapy faces challenges, including ethical concerns, high costs, and the need for long-term safety data. As research continues to advance, integrating gene therapy into comprehensive care models will require collaboration among healthcare providers, geneticists, and policymakers to address these challenges effectively^[61]^.

### Stem cell transplantation

Stem cell transplantation is another innovative approach that has transformed the treatment of various conditions, particularly hematologic diseases and certain solid tumors. By harnessing the regenerative properties of stem cells, this therapy aims to restore normal function in damaged tissues or organs. There are two primary typesofstemcellsused intransplantation: hematopoietic stem cells (HSCs), which give rise to blood cells, and mesenchymal stem cells (MSCs), which can differentiate into various cell types, including bone, cartilage, and fat cells. Stem cell transplantation is commonly used in treating conditions such as leukemia, lymphoma, and multiple myeloma. It can also be employed in regenerative medicine to repair damaged tissues in conditions like heart disease, spinal cord injury, and neurodegenerative disorders. Clinical studies have reported successful outcomes, with patients achieving remission or significant recovery after transplantation. The integration of stem cell transplantation into comprehensive care necessitates a multidisciplinary approach, involving oncologists, hematologists, transplant surgeons, and supportive care teams. This collaboration ensures that patients receive comprehensive assessments, appropriate pretransplant evaluations, and post-transplant follow-up care^[62]^.

### Enhancing patient support systems and resources

Patient support systems and resources are essential for helping individuals manage SCA effectively^[62]^. Future research should focus on developing and evaluating new support structures, such as online support platforms, patient advocacy programs, and community-based initiatives. These resources can offer patients emotional support, practical advice, and access to educational materials. Additionally, there is a need for research into the effectiveness of telehealth services for delivering care and support to patients who may have limited access to specialized healthcare providers. Exploring how these support systems can be improved and expanded will be crucial for enhancing patient care and engagement in disease management.

### Investigating the psychological impact of sickle cell anemia

Future research opportunities include investigating the long-term effects of SCA on mental health, including the prevalence of anxiety, depression, and stress among patients. Studies could focus on identifying factors that contribute to psychological distress and evaluating the effectiveness of various psychosocial interventions, such as counseling, support groups, and stress management techniques. Research in this area can lead to the development of targeted mental health support programs that address the specific needs of SCA patients and improve their overall well-being.

### Expanding genomic research for personalized medicine

Genomic research offers exciting opportunities for advancing personalized medicine in SCA management^[63]^. Future studies should focus on identifying genetic variations that influence disease severity, treatment responses, and complications. By exploring the genetic underpinnings of SCA, researchers can develop personalized treatment plans that are tailored to individual genetic profiles. This approach could lead to more effective and targeted therapies, as well as improved strategies for predicting disease progression and managing complications. Additionally, research into pharmacogenomics could help optimize drug therapies based on genetic factors, enhancing the precision of treatment regimens for SCA patients.

### Improving pain management strategies

Pain management remains a central challenge in SCA care, and there is a need for ongoing research into new pain management strategies^[64]^. Future research opportunities include exploring novel analgesic therapies, such as targeted drug delivery systems and pain-modulating agents. Studies could also investigate the effectiveness of multimodal pain management approaches, which combine medications, physical therapies, and behavioral interventions. Additionally, research into the biological mechanisms of pain in SCA can provide insights into new treatment options and improve current pain management practices. These efforts aim to reduce pain intensity, frequency, and impact on patients’ lives.

### Enhancing disease prevention and early intervention

Disease prevention and early intervention are crucial for improving outcomes in SCA management^[65]^. Future research should focus on developing strategies for early detection of complications and preventive interventions that can be implemented before symptoms arise. This includes exploring new screening methods, such as advanced imaging techniques or biomarker discovery, to identify risks for complications early. Additionally, research into preventive care practices, such as vaccination programs and lifestyle modifications, can help reduce the incidence of diseaserelated complications and improve patient health.

### Investigating healthcare delivery models for sickle cell anemia

Research into healthcare delivery models aims to improve the organization and efficiency of SCA care. Future studies could evaluate different care delivery models, such as patient-centered medical homes, integrated care teams, and care coordination programs. These models focus on improving access to care, streamlining services, and enhancing the patient experience. Research opportunities include assessing theeffectiveness of these models in achieving better health outcomes, reducing healthcare costs, and increasing patient satisfaction. By exploring and refining these models, researchers can contribute to the development ofmore effective and efficient caresystems forSCA patients.

### Addressing health disparities in sickle cell anemia care

Health disparities in SCA care are a significant issue, and future research should aim to address these inequalities. Studies can explore the socioeconomic factors that impact access to care, treatment adherence, and health outcomes for SCA patients. Research could focus on developing strategies to reduce disparities, such as improving access to quality care in underserved communities, increasing awareness about SCA, and advocating for policy changes. Addressing these disparities is essential for ensuring that all patients receive equitable care and have the opportunity to achieve optimal health outcomes.

### Exploring the role of technology in sickle cell anemia management

Technology has the potential to transform SCA management through innovative solutions for diagnosis, treatment, and patient support. Future research should explore the development and application of digital health tools, such as mobile health apps, wearable devices, and virtual care platforms. These technologies can offer patients new ways to monitor their health, manage their treatment regimens, and communicate with healthcare providers. Research opportunities include evaluating the effectiveness of these tools in improving disease management, enhancing patient engagement, and facilitating remote care. By leveraging technology, researchers can develop new methods for supporting patients and improving care outcomes.

## Conclusion

Managing emotional and physical stress in individuals with sickle cell anemia is paramount to improving their overall quality of life and health outcomes. Effective management strategies must address both dimensions of stress through a combination of pharmacological interventions, psychological support, and lifestyle modifications. The implementation of holistic approaches, such as cognitive-behavioral therapy, mindfulness practices, and support groups, can significantly enhance emotional well-being and resilience in patients. Moreover, addressing physical stressors through pain management techniques, preventive care, and tailored exercise programs plays a crucial role in reducing the frequency and severity of sickle cell crises.

## Data Availability

Not applicable as this a review.

## Research registration unique identifying number (UIN)

Not applicable as this a review.
